# Solid-State Synthesis for High-Tetragonality, Small-Particle Barium Titanate

**DOI:** 10.3390/ma17225655

**Published:** 2024-11-20

**Authors:** Tianyu Hao, Jing Shen, Qiaochu Peng, Jie Liu, Wenbin Hu, Cheng Zhong

**Affiliations:** 1 Key Laboratory of Advanced Ceramics and Machining Technology (Ministry of Education), Tianjin Key Laboratory of Composite and Functional Materials, School of Materials Science and Engineering, Tianjin University, Tianjin 300072, China; haotianyu909@163.com (T.H.); qcpeng@tju.edu.cn (Q.P.); wbhu@tju.edu.cn (W.H.); 2 Chongqing Newcent New Materials Co., Ltd., Chongqing 401147, China; shenjing@chinanewcent.com

**Keywords:** barium titanate, solid-state synthesis, tetragonality, size effects, ball milling, mechanochemical method

## Abstract

This study successfully synthesized high-tetragonality barium titanate (BaTiO_3_) particles with a small particle size by implementing ball milling in the solid-state synthesis of BaTiO_3_ and utilizing nanoscale raw materials. This study also addressed the issues of impurities and uneven particle size distribution that could exist in the synthesized BaTiO_3_ particles. The crystal structure, morphology, and particle size of the synthesized BaTiO_3_ particles have been meticulously analyzed and discussed through the use of techniques such as X-ray diffraction (XRD), scanning electron microscopy (SEM), and the laser particle size analyzer. BaTiO_3_ has been successfully synthesized, exhibiting a uniform particle size with an average diameter of 170 nm and a high tetragonality value of 1.01022. This new solid-state synthesis method provided insights to avoid the impact of “size effects” during the process of electronic device miniaturization.

## 1. Introduction

Barium titanate (BaTiO_3_) is a perovskite-type material widely used in multilayer ceramic capacitors (MLCCs) because of its high dielectric constant [1,2]. Among the various structures of BaTiO_3_, the tetragonal structure has gained significant attention for its distinct features [3,4,5]. Tetragonal BaTiO_3_ exhibits a significant property in the form of its tetragonal distortion of the structure cell, commonly referred to as tetragonality (*c/a*). This property is defined as the ratio of *a* and *c* lattice parameters [6]. Furthermore, tetragonality plays a crucial role in determining the dielectric properties of BaTiO_3_, as it has been positively correlated with such properties [3]. Thus, high tetragonality is highly desirable for achieving better dielectric properties in BaTiO_3_, which is particularly beneficial for its application in MLCCs.

In recent years, the miniaturization of MLCCs and electronic devices has become a prominent trend, leading to an increasing demand for electronic components with thin dielectric layers [7,8]. Therefore, the reduction in the particle size in BaTiO_3_ powders is a general trend. However, studies have indicated that the decrease of BaTiO_3_ particle size generally results in a reduction in the tetragonality of BaTiO_3_ particles, which is known as the “size effect” [4]. For example, Uchino et al. [9] observed that a high *c/a* of approximately 1.008 was sustained with the particle size of BaTiO_3_ being approximately 230 nm, but it decreased to approximately 1.002 at 130 nm. Therefore, it is difficult to prepare BaTiO_3_ particles that consider both high tetragonality and small particle size.

To this end, various synthesis methods have been made to prepare BaTiO_3_ with high tetragonality and small particle size. The hydrothermal method produces finely dispersed particles suited for thin dielectric layers [10], but the crystallographic structure may become cubic and not tetragonal because of the OH group incorporation. The synthesis of BaTiO_3_ from oxalates often has a complicated procedure. Kita et al. [11] used barium titanyl oxalate as a precursor to prepare BaTiO_3_ with well-dispersed particle size by the two-step thermal decomposition method. But the decomposition process lasted 5 h and the heat treatment lasted 16 h. Wada et al. [12] also obtained BaTiO_3_ particles by the two-step thermal decomposition method using BaTiO(C_2_O_4_)_2_·4H_2_O as the precursors. However, BaTiO(C_2_O_4_)_2_·4H_2_O was annealed in the O_2_ flow in the first step and the precursors were annealed in vacuum in the second step.

The solid-state synthesis method of BaTiO_3_ involves the calcination of a proportioned mixture of titanium dioxide (TiO_2_) and barium carbonate (BaCO_3_) [13,14]. This traditional method is known for its simplicity, generally resulting in high-tetragonality (*c/a* ratio) BaTiO_3_ products [15,16]. However, solid-state synthesis methods also have some limitations. The high-temperature calcination process has high energy consumption, and during the calcination process, the particle size distribution of BaTiO_3_ may be uneven, making it difficult to ensure its morphology. Impurities or incompletely reacted raw materials may also appear. These issues will all affect the electrical properties of BaTiO_3_ [15,17]. Therefore, some researchers are still committed to innovating and improving solid-state synthesis methods to achieve more efficient and environmentally friendly BaTiO_3_ powder synthesis processes.

In this research, the initial step involved analyzing BaTiO_3_ samples that were synthesized using the solid-state direct calcination method. Subsequently, ball treatments were applied to both the mixed raw materials and the barium titanate product. This approach aimed to investigate the impact of a two-step ball milling process on the synthesized barium titanate. Afterwards, the raw materials used for synthesis were replaced with nanoscale BaCO_3_ and nano-TiO_2_ with different particle sizes, and the influence of different particle sizes of raw materials on the synthesized barium titanate was discussed. These two methods effectively eliminated impurities that are prone to occur in solid-state synthesis and achieved a uniform and fine BaTiO_3_ particle size.

The solid-state synthesis method involved in this study successfully synthesized BaTiO_3_ particles with an average particle size (D50) of about 170 nm, exhibiting excellent uniformity and high tetragonality with a *c/a* near 1.01022. The synthesized barium titanate powder has important prospects for the miniaturization of electronic devices, as it maintains high tetragonality while ensuring uniform particle size.

## 2. Materials and Methods

### 2.1. Materials

Titanium dioxide (TiO_2_, 5–10 nm, 99.8%) was purchased from JiuDing Biotechnology, Shanghai, China. Titanium dioxide (TiO_2_, 25 nm, 99.8%) was purchased from Macklin Biochemical Technology, Shanghai, China. Titanium dioxide (TiO_2_, 40 nm, 99.8%) was purchased from Aladdin Biochemical Technology, Shanghai, China. Titanium dioxide (TiO_2_, 99%) was purchased from Meryer Chemical Technology, Shanghai, China. Barium carbonate (BaCO_3_, 0.5–1.5 μm, 99.8%) was purchased from Aladdin Biochemical Technology, Shanghai, China. Barium carbonate (BaCO_3_, 99% in 30–80 nm) was purchased from Maya Reagent. Ethanol (C_2_H_5_O, ≥99.8%) was bought from Macklin Biochemical Technology, Shanghai, China. All reagents were used directly after purchase.

### 2.2. Preparation of BaTiO_3_ Particles

**Figure 1:**
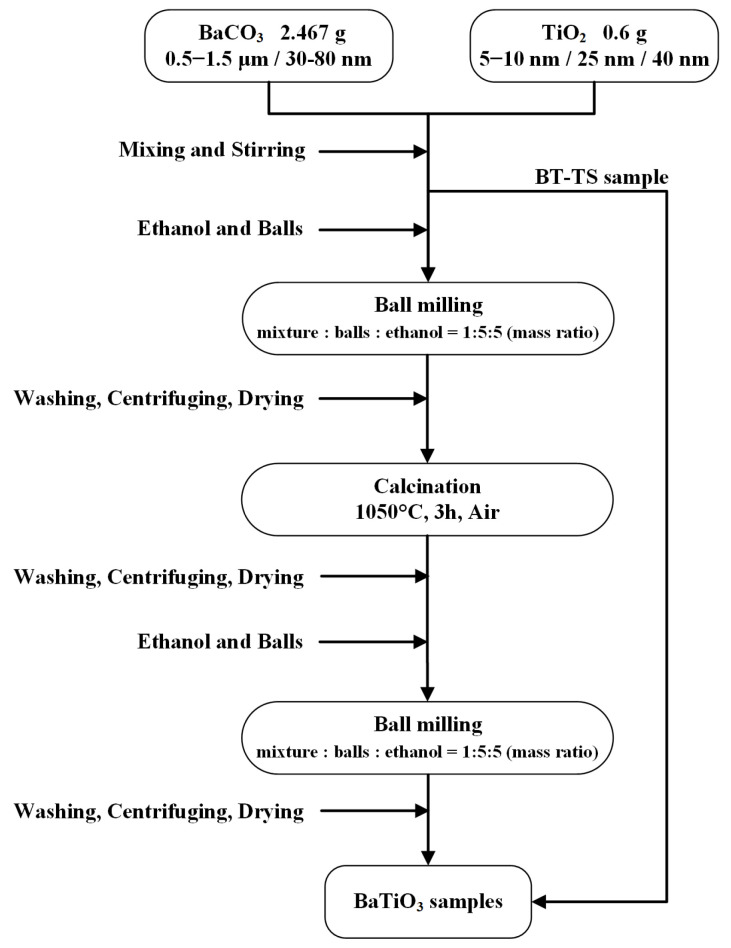
Flow chart for the preparation of BaTiO_3_ samples.

This solid-state synthesis method for BaTiO_3_ involves the use of two primary raw materials, titanium dioxide (TiO_2_) and barium carbonate (BaCO_3_). This study incorporated three different particle sizes of an anatase-type TiO_2_ (5–10 nm, 25 nm, and 40 nm) and two variations of BaCO_3_ (micrometer-scale and 30–80 nm scale). The raw materials were mixed in a stoichiometric molar ratio of Ba to Ti elements (Ba: Ti = 1:1), and for the preparation of the composite, 0.6 g of TiO_2_ and 2.467 g of BaCO_3_ were blended in a laboratory beaker. Figure 1 illustrates the detailed synthetic and fabrication process of BaTiO_3_ employed in this research.

The resulting amalgamation was then transferred to a 50 mL stainless steel ball milling jar, where it was processed using zirconium oxide grinding balls within an ethanol milieu. The mass ratio of raw materials to grinding balls to ethanol was set at 1:5:5, and the ball milling rotation speed was 240 rounds per minute (rpm).

After the initial ball milling pretreatment, the amalgamated mixture was transferred to alumina crucibles for the calcination process, which was conducted under ambient air conditions at a temperature of 1050 °C for 3 h. Following calcination, the raw BaTiO_3_ product was pulverized and subjected to a second ball milling, employing identical parameters as in the prior operation. The solid–liquid mixture was then transferred into a centrifuge tube and subjected to a single centrifugation cycle. The resulting product was subjected to successive rinses and an acetic acid solution. Following the decantation of the supernatant, the residual material was desiccated in an oven set to 80 °C for 12 h. Finally, the resulting desiccated white BaTiO_3_ solid was comminuted into a finely powdered form.

### 2.3. Characterization of BaTiO_3_ Particles

The crystal phase and lattice constants of BaTiO_3_ particles were analyzed using X-ray diffraction instrument (XRD, D8 Advanced, Bruker AXS GmbH, Karlsruhe, Germany) with Cu K_α_ radiation at 40 kV and 40 mA. The morphology of BaTiO_3_ was analyzed using a scanning electron microscope (SEM, Hitachi S4800, Tokyo, Japan). The particle size distribution and average particle sizes (D50) of the BaTiO_3_ powders were determined using a particle size analyzer (Mastersizer 2000, Malvern Instruments Ltd., Worcestershire, UK). The measurement range spanned from 0.02 μm to 2000 μm, and the ethanol was used as the dispersant. The element distribution was analyzed using energy dispersive spectrometer (EDS) equipment (Ultim Max, Oxford Instruments, Oxford, UK) and X-ray photoelectron spectroscopy (XPS) equipment (K-Alpha, Thermo Scientific, Waltham, MA, USA).

## 3. Results and Discussion

### 3.1. Analysis of Barium Titanate Synthesized by Direct Calcination

**Figure 2:**
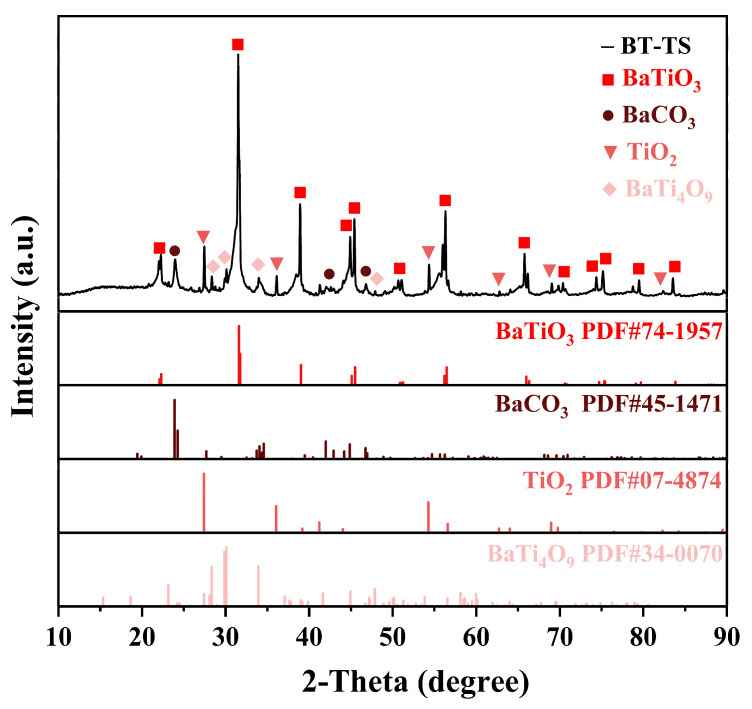
XRD pattern of BaTiO_3_ sample BT-TS.

Figure 2 displays the X-ray diffraction pattern of the BaTiO_3_ sample (BT-TS), synthesized via the traditional solid-state synthesis method, which involved calcining a mixture of micrometer-scale BaCO_3_ and TiO_2_ precursors at 1050 °C and 3 h under an air atmosphere. In Figure 2, the main diffraction peaks aligned with the tetragonal *P4mm* space group characteristic of BaTiO_3_ (JCPDS 74-1957), confirming that BaTiO_3_ has been synthesized. However, upon the comparative analysis with the standard PDF card, impurities were identified, including BaTi_4_O_9_ (JCPDS 34-0070), unreacted TiO_2_ (JCPDS 07-4874), and BaCO_3_ (JCPDS 45-1471) raw materials.

In solid-state reactions, the intermediate processes of barium titanate synthesis can be summarized as follows [18]. The first step is to directly decompose the BaCO_3_ raw material powder into BaO and CO_2_ gas in a high-temperature reaction. Part of BaCO_3_ reacts with the titanium source TiO_2_ to generate the product BaTiO_3_, while the remaining BaCO_3_ generates the Ba_2_TiO_4_ phase, which is expressed as the following reaction:

BaCO_3_ → BaO + CO_2_ (1)

BaCO_3_ + TiO_2_ → BaTiO_3_ + CO_2_ (2)

2BaCO_3_ + TiO_2_ → Ba_2_TiO_4_ + 2CO_2_ (3)

In this step, the Gibbs free energy of BaCO_3_ decomposition is the lowest, making it the main reaction in this part. In the second step, the BaO generated by the first reaction will simultaneously generate the Ba_2_TiO_4_ phase, which can be expressed as

BaO + TiO_2_ → BaTiO_3_ (4)

2BaO + TiO_2_ → Ba_2_TiO_4_ (5)

This indicates that there will be products generated by the non-1:1 combination of Ba and Ti during the calcination process. It also explains the impurities in Figure 2, which are the remaining raw materials that have not fully reacted and the source of BaTi_4_O_9_ generated due to an insufficient reaction. In order to eliminate impurities and synthesize pure barium titanate powder to solve the impurity problem in the solid-state method, the next section of research on the ball milling treatment is introduced from the perspective of the binding and mixing of raw materials before calcination.

### 3.2. The Effect of Ball Milling Treatment on the Synthesized Barium Titanate

In order to investigate the effects of two-step ball milling on the synthesis process, four different samples were synthesized. The four different samples include the following:

- BT0-0 is BaTiO_3_ sample synthesized without any ball milling treatment.
- BT0-1 is BaTiO_3_ sample synthesized solely for ball milling of raw BaTiO_3_.
- BT1-0 is BaTiO_3_ sample synthesized solely for ball milling of raw materials.
- BT1-1 is BaTiO_3_ sample synthesized by both two-step ball milling treatment.

**Figure 3:**
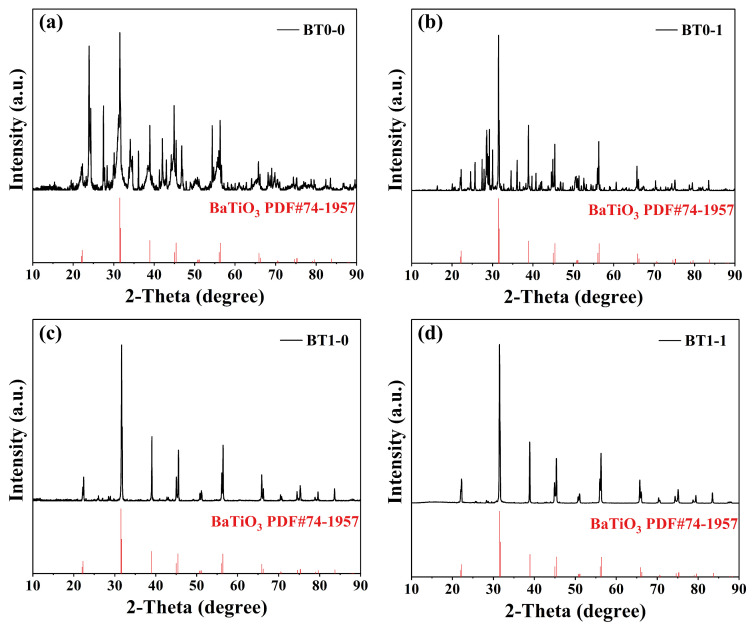
Main XRD patterns of BaTiO_3_ samples synthesized with and without ball milling: (**a**) BT0-0, (**b**) BT0-1, (**c**) BT1-0, and (**d**) BT1-1.

The raw materials for these four samples are TiO_2_ and micrometer-sized BaCO_3_, and the calcination conditions are all 1050 °C and 3 h. The mass ratio of raw materials to grinding balls to ethanol was set at 1:5:5, and the ball milling process had a rotation speed of 240 rpm. The XRD patterns of the four BaTiO_3_ samples synthesized with and without ball milling are shown in Figure 3. Firstly, for the samples BT0-0 and BT0-1, the XRD patterns clearly show not only the characteristic peaks of the tetragonal *P4mm* space group BaTiO_3_ (JCPDS 74-1957) that was mainly synthesized but also a significant number of peaks corresponding to impurities. Similar to BT-TS, these two samples were not subjected to ball milling of the raw materials. Comparing BT0-0 with BT1-0, it is not difficult to find that after ball milling of the raw materials followed by synthesis, the characteristic peaks of the tetragonal *P4mm* space group BaTiO_3_ (JCPDS 74-1957) in the XRD pattern of BT1-0 are very distinct and basically consistent with the PDF standard cards. This indicates that ball milling of the raw materials indeed promotes the synthesis reaction and makes the reaction more complete, with impurities essentially disappearing. However, it is still possible to observe minor impurity peaks in the range of 2*θ* = 25°−30°, which is noteworthy. Lastly, for the sample BT1-1, the standard PDF card comparison result is essentially consistent with that of BT1-0, and there are still minor impurity peaks in the range of 2*θ* = 25°−30°, indicating that the simple introduction of ball milling cannot completely eliminate the impurity issues in solid-state synthesis methods, and further analysis and research from other perspectives are necessary.

On the other hand, the impact of the introduction of ball milling on the tetragonality of the synthesized BaTiO_3_ is also very much worth noting. Therefore, based on the analysis and calculation of the crystal structures of the four samples in the software JADE 9.0, different tetragonalities *c/a* were obtained, as shown in Table 1. From Table 1, it can be seen that the tetragonality of the samples is all around 1.01 except BT0-1, which on one hand indicates that the tetragonality of BaTiO_3_ synthesized by the solid-state method is indeed good. The tetragonality is affected due to the fact that BT0-0 and BT0-1 did not produce pure BaTiO_3_. On the other hand, by comparing the tetragonality of BT1-0 and BT1-1, it can be seen that ball milling of the BaTiO_3_ has no negative impact on the tetragonality.

**Figure 4:**
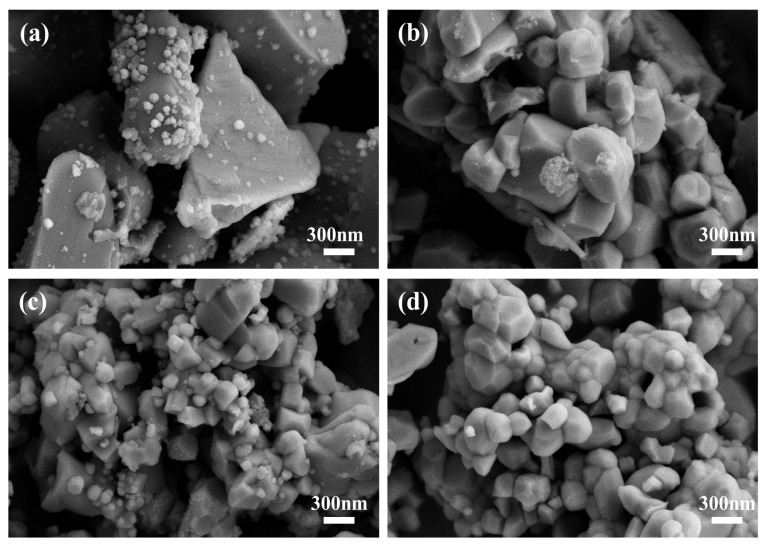
SEM images of BaTiO_3_ samples synthesized with and without ball milling: (**a**) BT0-0, (**b**) BT0-1, (**c**) BT1-0, and (**d**) BT1-1.

Figure 4 presents the SEM micrographs of the aforementioned four samples. Firstly, from a cross-sectional comparison perspective, it is observed that the samples BT0-0 and BT0-1 share some common morphological characteristics. Specifically, fine particles can be observed adhering to larger particles in both samples. However, the agglomeration of particles in the BT0-1 sample is significantly reduced, indicating that the ball milling of BaTiO_3_ has improved the interparticle interactions and enhanced the dispersion of the particles.

Secondly, through a longitudinal comparison, it can be noted that the samples BT1-0 and BT1-1, in contrast to the samples BT0-0 and BT0-1, no longer exhibit the phenomenon of fine particles adhering to larger particles. This change may be due to the ball milling process, which allows for a more thorough mixing of the raw materials, leading to an increase in the surface energy, Gibbs free energy, and activation energy of the crystals. Consequently, in the subsequent synthesis reaction, the interactions between the raw materials are more complete, and the reaction process is more thorough and in-depth.

When the cumulative particle size distribution percentage reaches 10%, 50%, and 90%, the sample particle sizes are represented as D10, D50, and D90, respectively, indicating that particles smaller than these values account for 10%, 50%, and 90%. These three values provide a relatively intuitive view of the particle size distribution of the powder [19]. D10 and D90 represent the fine and coarse diameters of the powder, respectively, while D50 is the median particle size of the powder. The particle size distribution and average particle sizes (D50) of the BaTiO_3_ powders were determined using a particle size analyzer (Mastersizer 2000, Malvern Instruments Ltd., Worcestershire, UK).

The D10, D50, and D90 data recorded in Table 1 provide us with a quantitative perspective to evaluate the impact of ball milling on the particle size distribution of BaTiO_3_ titanate samples. These data represent the particle sizes corresponding to 10%, 50%, and 90% of the particles in the sample, which are important parameters for describing the characteristics of the particle size distribution.

For the two samples, BT0-0 and BT0-1, it can be observed that the BT0-1 sample, which underwent ball milling of BaTiO_3_, has reduced values in all three parameters of D10, D50, and D90. Specifically, the average particle size (D50) of BT0-1 decreased from 6.125 μm to 3.551 μm, and the D10 and D90 values also decreased accordingly, indicating that the overall particle size of the sample was significantly reduced after ball milling. This result is consistent with the morphological changes observed by SEM; that is, ball milling can effectively reduce particle size and improve particle dispersion.

More notably, the particle size distribution data of the BT1-1 sample is significant. The average particle size of the BT1-1 sample is 0.171 μm, and its particle size distribution is very concentrated, with most particles ranging from 0.131 μm to 0.226 μm in size. This result indicates that the BT1-1 sample, after the second ball milling process, not only has an extremely small particle size but also a highly uniform particle size distribution. This demonstrates that ball milling plays a key role in controlling the particle size distribution of barium titanate powder, especially in achieving a highly uniform distribution.

In summary, the D10, D50, and D90 data presented in Table 1 allow for a quantitative analysis and comparison of the effects of different ball milling treatments on the particle size distribution of BaTiO_3_ samples. These data, combined with SEM observations, provide us with a comprehensive perspective to understand the specific effects of ball milling on the morphology and particle size distribution of BaTiO_3_ samples. These analyses lead to the conclusion that ball milling is an effective method for optimizing the particle size distribution of barium titanate powder, potentially enhancing its final performance.

The ball milling treatment had a significant positive effect on improving the morphology and particle size distribution of barium titanate samples. In particular, after ball milling of BaTiO_3_, the particle size distribution of the barium titanate samples was significantly optimized. The particle sizes became more uniform and concentrated. Compared with the samples that were not subjected to ball milling, there was a noticeable reduction in the average particle size, which was clearly demonstrated in both the SEM images and the particle size distribution data. The decrease in parameters such as D10, D50, and D90 further confirmed the effectiveness of the ball milling treatment.

### 3.3. The Influence of Raw Materials with Different Particle Sizes on the Synthesis of Barium Titanate

In this part, the BaTiO_3_ samples were synthesized using micrometer-sized (BC-μm) and 30−80 nm sized BaCO_3_ (BC-nm). The calcination conditions are all 1050 °C and 3 h. The mass ratio of raw materials to grinding balls to ethanol was set at 1:5:5, and the ball milling process had a rotation speed of 240 rpm.

**Figure 5:**
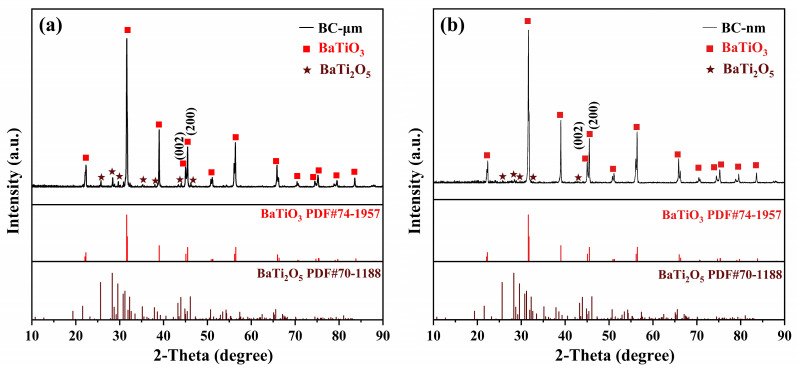
XRD patterns of BaTiO_3_ samples synthesized by BaCO_3_ with different particle sizes: (**a**) BC-μm and (**b**) BC-nm.

Figure 5 presents the XRD pattern of BC-μm and BC-nm. For both BC-μm and BC-nm samples, the XRD results indicate that the main diffraction peaks correspond to the characteristics of the tetragonal *P4mm* space group of BaTiO_3_ (JCPDS 74-1957), once again confirming the successful synthesis of BaTiO_3_. Compared to the BT-TS sample in Figure 2, the absence of peaks for unreacted TiO_2_ and BaCO_3_ is very evident, indicating that the ball milling process can effectively promote a comprehensive reaction of the raw materials, thereby significantly reducing the presence of unreacted raw materials. Moreover, the peaks for BaTi_4_O_9_ have also completely disappeared, further demonstrating the significant effect of the ball milling process in improving reaction efficiency and reducing impurities.

Despite undergoing two stages of ball milling treatment, minor impurities are still observable in the XRD results of both BC-μm and BC-nm near 25° and 28°. By comparing with the standard PDF cards, these peaks are identified as BaTi_2_O_5_ (JCPDS 70-1188). Compared to the BaTi_4_O_9_ present in BT-TS, where the ratio of Ba to Ti elements is 1:4, BaTi_2_O_5_ exhibits a higher ratio of 1:2, indicating a higher degree of reaction between Ba and Ti elements. Although the BC-μm and BC-nm samples have improved in terms of impurities and element ratios compared to the BT-TS sample, it is noteworthy that the element ratio of Ba to Ti has not yet reached the ideal 1:1 ratio. This suggests that the micro-level interaction between Ba and Ti still needs to be enhanced, and therefore the BC-μm and BT40 samples are not entirely composed of pure BaTiO_3_. This finding suggests that while the ball milling treatment played a positive role in reducing impurities during the calcination process, completely eliminating the presence of impurities remains a challenge.

In the XRD results of BC-μm and BC-nm, the split peaks near 2*θ* = 45° for the (002) and (200) crystal planes provide key evidence for the successful synthesis of the tetragonal phase of BaTiO_3_. The symmetrical shape and non-split state of these peaks further confirm the tetragonal structure of the BC-μm and BC-nm samples. Crystallographic calculations using the JADE software show a *c/a* ratio of 1.00964 for the BC-μm sample and a ratio of 1.00986 for the BC-nm sample. On the other hand, it also suggests that the use of nano-sized BaCO_3_ rarely has an impact on the tetragonality of the synthesized barium titanate.

Generally, TiO_2_ with a smaller particle size, due to its larger specific surface area, reacts more rapidly with BaCO_3_, which helps to improve the efficiency of barium titanate synthesis. The particle size of titanium dioxide may affect the final crystal structure of barium titanate. The particle size distribution of titanium dioxide has a direct impact on the particle size distribution of barium titanate powder. A uniform particle size of titanium dioxide is conducive to obtaining a barium titanate powder with a uniform particle size distribution, which is particularly important for the preparation of high-performance electronic ceramic materials. To thoroughly investigate and address the issue of residual BaTi_2_O_5_ impurities in the BC-μm and BC-nm samples, and to fully understand the specific effects of different TiO_2_ particle sizes on the crystal structure and morphology of BaTiO_3_, this section simultaneously uses TiO_2_ with different particle sizes (5–10 nm, 25 nm, and 40 nm) to synthesize three samples, BT5-10, BT25, and BT40, based on the use of nano-sized BaCO_3_ as the raw material. The calcination conditions are all 1050 °C and 3 h. The mass ratio of raw materials to grinding balls to ethanol was set at 1:5:5, and the ball milling process had a rotation speed of 240 rpm.

**Figure 6:**
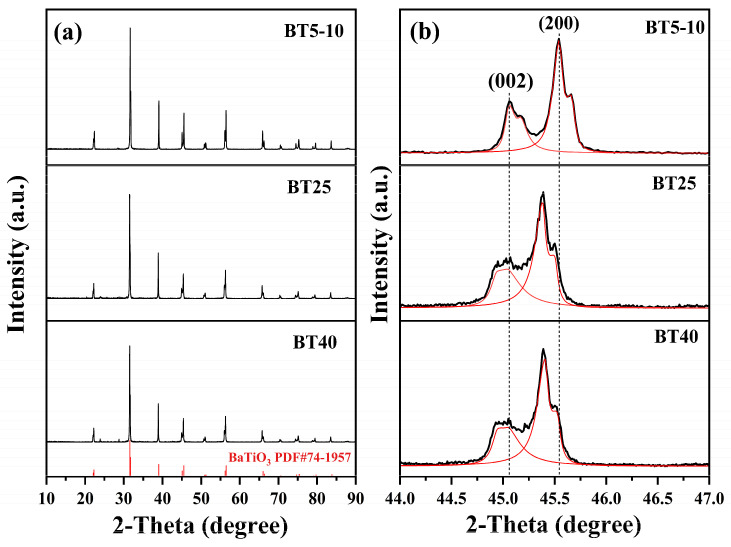
XRD patterns of BaTiO_3_ samples synthesized using different sizes of TiO_2_ precursors: (**a**) XRD patterns of BT5-10, BT25, and BT40; (**b**) XRD patterns of BT5-10, BT25, and BT40 after amplification.

Figure 6, from top to bottom, shows the XRD and the locally enlarged XRD diagrams of the three samples. In Figure 6a, the XRD results of all three samples are consistent with the standard PDF card (JCPDS 74-1957), confirming that the synthesized crystals are BaTiO_3_. This result indicates that nano-sized raw materials with different particle sizes can effectively be used to produce BaTiO_3_ with a stable structure. Compared with BT40, no impurity peaks were observed in the XRD diagram of BT5-10, confirming that the introduction of ball milling technology and the use of nano-sized raw materials can effectively eliminate impurities such as BaTi_4_O_9_ and BaTi_2_O_5_. The results in Figure 6b further reveal that all three samples have split peaks near 2*θ* = 45° for the (002) and (200) crystal planes, which provides key evidence for the successful synthesis of the tetragonal phase of BaTiO_3_. In Figure 6b, the red curve represents the fitting of the split peaks at 45° for different samples. The symmetrical shape and non-split state of these peaks further confirm the existence of the tetragonal structure. It is noteworthy that the splitting between these two peaks is most pronounced in the BT5-10 sample, indicating that it has the best tetragonality.

**Figure 7:**
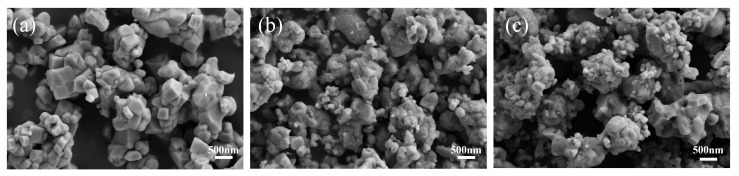
SEM images of BT5-10, BT25, and BT40: (**a**) SEM image of BT5-10; (**b**) SEM image of BT25; (**c**) SEM image of BT40.

Figure 7 presents the SEM images of the three samples, providing intuitive information about the sample morphology. As shown in Figure 7a, the BT5-10 sample exhibits the best particle dispersion and distribution among all the samples. This may be attributed to the smaller particle size of TiO_2_, which provides a larger specific surface area and higher reactivity, thus promoting more uniform mixing and dispersion. The BT25 sample in Figure 7b, while also showing improved particle dispersion, has slightly less uniformity compared to BT5-10. In contrast, the BT40 sample in Figure 7c displays a noticeable particle agglomeration and poorer dispersion.

According to the data presented in Table 2, the *c/a* ratios for the three samples are 1.01022, 1.00941, and 1.00924, respectively. From these data, it is evident that the BT5-10 sample exhibits the best tetragonality. This result further confirms that the use of nano-sized raw materials has a significant impact on enhancing the tetragonal nature of BaTiO_3_. Due to their higher specific surface area and reactivity, nano-sized raw materials can provide a more uniform and refined reaction environment during the synthesis process, which is conducive to forming a more complete crystal structure and improving the tetragonality of BaTiO_3_.

Furthermore, the study also found that, with the condition that BaCO_3_ is nano-sized, and other synthesis parameters remain unchanged after the two-step ball milling treatment, the smaller the particle size of nano-TiO_2_, the better the tetragonal nature of the synthesized BaTiO_3_. This may be because the smaller particle size of TiO_2_ is beneficial for improving the homogeneity of the raw material mixture, reducing particle agglomeration, and thus forming more uniform and well crystallographically oriented barium titanate particles during the sintering process. BaTiO_3_ tends to form on the surface of TiO_2_ particles. When a surface layer of BaTiO_3_ is formed, the kinetics are controlled by the diffusion of barium and oxygen ions through this layer into the original TiO_2_ phase. Since these ions are in excess on the surface layer, the Ba_2_TiO_4_ phase is typically formed initially.

Due to continuous diffusion, the interaction of Ba_2_TiO_4_ and TiO_2_ gradually formed uniform BaTiO_3_ particles. It suggests that fine anatase TiO_2_ with a low density and a large surface area enhances the formation of BaTiO_3_ by reducing the activation energy, which is consistent with our findings.

Table 2 also lists the D10, D50, and D90 values for the three samples. It is observed that the average particle size of all three samples is around 170 nm.

Due to the fact that sample BT5-10 not only ensures a high degree of tetragonality (1.01022) but also maintains a relatively small particle size (170 nm), specific elemental composition analysis was conducted on this sample to verify the purity of barium titanate and ensure uniform elemental distribution.

**Figure 8:**
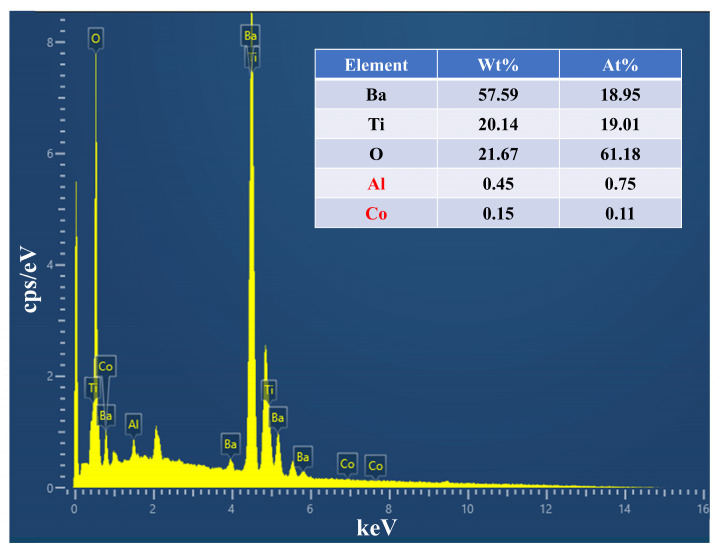
EDS images of BT5-10.

The energy-dispersive spectroscopy (EDS) data and quantitative elemental analysis for BT5-10 are presented in Figure 8. The sample exhibits an atomic composition of Ba (18.95%), Ti (19.01%), and O (61.18%), which approximates the 1:1:3 ratio characteristic of the stoichiometry of BaTiO_3_ [20].

**Figure 9:**
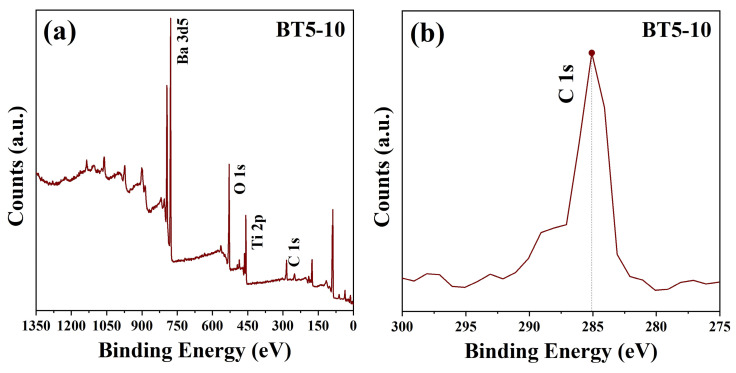
XPS images of BT5-10: (**a**) full spectra XPS of BT5-10; (**b**) High resolution XPS spectra of C 2*p*.

X-ray photoelectron spectroscopy (XPS) was conducted on the BT5-10 sample to achieve a more nuanced understanding of its elemental composition. The XPS results of BT5-10 are shown in Figure 9.

Figure 9a illustrates the high-resolution XPS spectrum of BT5-10. The distinct peaks observed at binding energies between 700 and 800 eV are assigned to the Ba 3*d*_5/2_ and Ba 3*d*_3/2_ levels, indicative of the perovskite structure characteristic of BaTiO_3_ [21].

The peaks in the vicinity of 450 eV are associated with the Ti 2*p*_3/2_ and Ti 2*p*_1/2_ transitions, which are diagnostic of the Ti^4+^ oxidation state. The absence of a lower binding energy satellite peak associated with the Ti 2*p*_3/2_ signifies that titanium ions are exclusively present in the oxidation state. This observation further supports the tetragonal structure of the material and indicates the absence of Ti vacancy defects in the synthesized samples [21].

Figure 9b displays the carbon peak C 1*s* from the XPS results of the BT5-10 sample. It is evident that the carbon peak is distributed very close to 285 eV, indicating that the sample does not contain impurity carbon but rather suggests the presence of adventitious carbon, likely introduced during the testing process as amorphous organic carbon [22].

Table 3 presents a comparison of particle size and tetragonality of barium titanate samples synthesized by different synthetic methods.

Based on a comprehensive analysis of the experimental data presented in this paper, it can be concluded that, provided that BaCO_3_ is nano-sized, the particle size of nano-TiO_2_ has a significant impact on the tetragonal nature of the synthesized BaTiO_3_ through a two-step ball milling process, with other synthesis parameters remaining constant. Specifically, using nano-TiO_2_ with a smaller particle size as the raw material can more effectively promote the formation of the tetragonal phase of BaTiO_3_, thereby enhancing the material’s tetragonal nature.

In this study, the BT5-10 sample synthesized using 5–10 nm TiO_2_ as the raw material demonstrated the best particle dispersion and distribution among all the compared samples, with an average particle size of 170 nm and a tetragonal nature of 1.01022.

## 4. Conclusions

In this study, a novel solid-state synthesis method was employed to prepare barium titanate (BaTiO_3_) powder, which is characterized by its high tetragonal nature, small particle size, and uniform distribution. The novel synthesis method primarily involves the use of small-sized raw materials and the ball milling treatment for both the raw materials and the coarse products.

Comprehensive characterizations, including XRD, SEM, LDM, EDS, and XPS, were conducted to evaluate the synthesized BaTiO_3_ powder, leading to the following conclusions:

(1) Ball milling treatment does not have a negative impact on the tetragonality. It resulted in a more homogeneous mixture of the raw materials, which helped to prevent the appearance of impurities after sintering.
(2) Ball milling treatment has a significant positive effect on improving the morphology and particle size distribution of BaTiO_3_.
(3) The replacement with nano-sized BaCO_3_ did not significantly affect the tetragonality or improve the particle size distribution and morphology of the synthesized BaTiO_3_.
(4) The particle size of nano-TiO_2_ has a significant impact on the tetragonality of BaTiO_3_. Generally, using smaller-sized nano-TiO_2_ as a raw material can more effectively promote the formation of the tetragonal phase of BaTiO_3_.

In this study, the BT5-10 sample synthesized using 5–10 nm TiO_2_ as the raw material showed the best particle dispersion and distribution among all the compared samples, with an average particle size of 170 nm and a tetragonal nature of 1.01022. This meets the contemporary electronic industry’s demand for high tetragonal nature, small, and uniformly sized BaTiO_3_ particles and also addresses some of the issues present in the solid-state method.

## Figures and Tables

**Table 1:** *c/a* and distributions of particle sizes.

Sample	*c/a*	D_10_ (μm)	D_50_ (μm)	D_90_ (μm)
BT0-0	1.01138	0.965	6.125	20.179
BT0-1	1.00586	0.648	3.551	15.214
BT1-0	1.01049	0.134	0.182	2.620
BT1-1	1.01017	0.131	0.171	0.226

**Table 2:** *c/a* and distributions of particle sizes.

Sample	*c/a*	D10 (μm)	D50 (μm)	D90 (μm)
BT5-10	1.01022	0.129	0.170	0.224
BT25	1.00941	0.132	0.177	0.253
BT40	1.00924	0.134	0.174	0.232

**Table 3:** Research results of synthesized BaTiO_3_.

Researchers	Heat Treatment (°C)	Particle Size (nm)	Tetragonality (*c/a*)	Method
Maison et al. [23]	1100	392	1.0098	Catecholate process
Kown et al. [24]	1000	243	1.0074	Hydrothermal
	1000	326	1.0105	
Tihtih et al. [25]	800	26.15	1.0021	Sol–gel process
	900	29.65	1.0054	
	1000	32.46	1.0063	
Clabel et al. [26]	1000	240	1.0060	Solid-state reaction
	1100	360	1.0081	
Xiaoxiao Pang et al. [27]	-	82	1.0088	Hydrothermal
Tingting Wang et al. [28]	-	near 160	near 1.00895	Hydrothermal
This work	1050	170	1.01022	Solid-state reaction

## Data Availability

Data are contained within the article.
